# Role of GSK-3β Inhibitors: New Promises and Opportunities for Alzheimer’s Disease

**DOI:** 10.34172/apb.2023.071

**Published:** 2023-01-23

**Authors:** Suggala Ramya Shri, Suman Manandhar, Yogendra Nayak, K Sreedhara Ranganath Pai

**Affiliations:** Department of Pharmacology, Manipal College of Pharmaceutical Sciences, Manipal Academy of Higher Education, Manipal -576104, India.

**Keywords:** Alzheimer’s disease, Allosteric inhibitors, Glycogen synthase kinase-3beta, ATP-competitive inhibitors, Proinflammatory cytokines, GSK-3β drug binding pockets

## Abstract

Glycogen synthase kinase-3 (GSK-3) was discovered to be a multifunctional enzyme involved in a wide variety of biological processes, including early embryo formation, oncogenesis, as well cell death in neurodegenerative diseases. Several critical cellular processes in the brain are regulated by the GSK-3β, serving as a central switch in the signaling pathways. Dysregulation of GSK-3β kinase has been reported in diabetes, cancer, Alzheimer’s disease, schizophrenia, bipolar disorder, inflammation, and Huntington’s disease. Thus, GSK-3β is widely regarded as a promising target for therapeutic use. The current review article focuses mainly on Alzheimer’s disease, an age-related neurodegenerative brain disorder. GSK-3β activation increases amyloid-beta (Aβ) and the development of neurofibrillary tangles that are involved in the disruption of material transport between axons and dendrites. The drug-binding cavities of GSK-3β are explored, and different existing classes of GSK-3β inhibitors are explained in this review. Non-ATP competitive inhibitors, such as allosteric inhibitors, can reduce the side effects compared to ATP-competitive inhibitors. Whereas ATP-competitive inhibitors produce disarrangement of the cytoskeleton, neurofibrillary tangles formation, and lead to the death of neurons, etc. This could be because they are binding to a site separate from ATP. Owing to their interaction in particular and special binding sites, allosteric ligands interact with substrates more selectively, which will be beneficial in resolving drug-induced resistance and also helpful in reducing side effects. Hence, in this review, we focussed on the allosteric GSK-3β inhibitors and discussed their futuristic opportunities as anti-Alzheimer’s compounds.

## Introduction

 Glycogen synthase kinase-3 (GSK-3) is a serine-threonine protein kinase involved in glycogen synthesis.^1^ GSK-3 was discovered in the 1980s reported attenuating glycogen synthase with its kinase activity.^2^ GSK-3 was discovered to be an enzyme with a multifunctional role that is concerned with a wide variety of biological processes such as early embryo formation in fruit flies and frogs, neurodegenerative disease, oncogenesis, and cell death.^3^ GSK-3β plays a role in the initiation and progression of a variety of health disorders, including diabetes, inflammation, Alzheimer’s disease (AD),^4^ cancer, bipolar disorder, Huntington’s,^5^ and Parkinson’s disease. As a result, GSK-3β is widely regarded as a promising target for therapeutic use. AD is a neurodegenerative disorder with clinical symptoms like progressive memory loss, cognitive impairment, executive dysfunction, and behavioral changes.^6^ However, the pathogenesis and etiology of AD are not known completely.

 The average life expectancy is three to nine years after the diagnosis of AD. At present, the FDA-approved drugs for AD treatment include NMDA receptor antagonists (memantine) and acetylcholinesterase inhibitors (rivastigmine, galantamine, donepezil,). These medications are only prescribed for the prevention of AD symptoms.^7^ Most of the available treatments have failed to demonstrate effectiveness, necessitating novel approaches to be considered.^8^ The efficacy of drugs varies from person to person and with temporary effect.^9^ There is still a need for disease-modifying therapeutics as there is a lack of success in the pharmacotherapy of AD.^10^ However, it is currently unclear what strategies the medication employs to achieve its therapeutic results.^11^

 The most prevalent form of dementia, according to World Health Organization (WHO) reports, accounts for about 60-70% of cases, in AD. WHO ranked AD as the seventh leading cause of death in 2019.^12^ The population of America aged 65 and up is expected to be 88 million by 2050. AD in India is approximated to rise to 6.35 million by the year 2025.^13^ In India, 14.2% of people are estimated to have AD by the year 2020. While there is no cure for this disease, treatment in the early stages could be more effective.

 Amyloid peptide precursor protein (APP) fragmentation leads to the formation of Amyloid beta. The Aβ aggregate forms oligomers, high-order polymerized structures that are toxic to neurons. Hyperphosphorylation of tau protein leads to the formation of neurofibrillary tangles in the main cytoskeleton. Aβ peptide favors phosphorylation of tau by GSK3β activation. Aβ peptide targets the Wnt pathway or insulin signaling pathways then increases the level of activated GSK-3β protein and, finally, tau phosphorylation.^14,15^ Hence, GSK-3β inhibitors may be used as therapeutic agents.^16^ Despite the success of in vivo and in vitro trials, clinical studies are yet to confirm its benefits.^17^ In this review, we have compiled different dimensions of GSK-3β inhibitors. A different class of GSK-3β inhibitors has been published in the literature, however, most of them are competitive ATP inhibitors with low specificity and undesirable side effects.^18,19^ Non-ATP competitive inhibitors, such as allosteric inhibitors, have immense potential to reduce risk by binding to a site separately from ATP.^20^ These compounds are commonly known to have better therapeutic effects in clinical applications.^21^ Today, allosteric is widely accepted as a natural property of all dynamic proteins of biological significance.^22^ Allosteric inhibitors, in particular, have a novel mechanism for modulating GSK-3β. Hence this review incorporates the importance and different known allosteric GSK-3β inhibitors.

## GSK-3 and its physiological importance

 GSK-3 is a SER/Thr kinase that is originally found in mammals, and homologs of GSK-3 have been found in all eukaryotes.^23,24^ In mammals, the GSK-3β and GSK-3α are two strongly homologous variants of GSK-3, encoded by separate genes. Humans have GSK-3α and GSK-3β, located on chromosome 19 and chromosome 3, respectively. GSK-3α shares 85% sequence of amino acid similarity, including 98% sequence shared by GSK-3β. GSK-3α encodes a 51-kilodalton protein, while GSK-3β encodes a 47-kilodalton protein, respectively. GSK-3α holds a large glycine-rich N-terminal region but is absent in GSK-3β. Protein kinase-C was reported to phosphorylate GSK-3β. Akt3, Akt1, and Akt phosphorylate GSK-3β, but Akt2 and IκB-related kinase-1 also phosphorylate GSK-3α.^25^

 GSK-3β is well understood and strongly expressed in the CNS, cortex, and nerves, whereas hardly visible in astrocytes. Neurogenesis, neuronal proliferation, differentiation, and survival are all regulated by GSK-3β in the developing brain.^26^ It is also involved in diseases like peritonitis, renal dysfunction, arthritis, hepatotoxicity associated with endotoxemia, colitis, and endotoxin shock. Deletion of GSK-3β leads to embryonic lethality on E16 (from the mice on the 16th day of gestation) that cannot be rescued by GSK-α.^27^ GSK-3 is a central kinase; it is also involved in the pathological phosphorylation of the microtubule-binding protein tau. The beta isozyme is required for development.^28^ GSK-3β activity is regulated through site-specific phosphorylation, and it is constitutively active in the resting cells. Phosphorylation at tyrosine 216 (Tyr216) is needed for full GSK-3β action, while phosphorylation at serine 9 (Ser9) inhibits the GSK-3β activity.

 The substrates of GSK-3β can be divided mainly into three groups, namely, (i) metabolic/signaling proteins: including acetyl CoA carboxylase, APP, cyclin D1, eukaryotic initiation factor-2B, glycogen synthase, ATP citrate lyase, cyclic AMP-dependent protein kinase, nerve growth factor receptor, Axin, protein phosphatase-1, insulin receptor substrate-1(IRS-1), protein phosphatase inhibitor-2, myelin basic protein, APC tumor suppressor protein and pyruvate dehydrogenase; (ii) structural proteins include microtubule-associated protein-2 (MAP-2), microtubule-associated protein-1B (MAP-1B), neural cell adhesion protein, neurofilaments, spindle-associated protein astrin, tau and ninein, and (iii) GSK-3β-targeted transcription factors: include AP-1 (also known as Jun family), β-catenin, Myc, NFAT, HSF-1, NF-κB, C/EBPα, CREB, and glucocorticoid receptor. The well-known substrate for GSK-3β is probably the β-catenin, which is critically involved in the Wnt signaling, in the development of neural pathways.

## Overview of GSK-3β structure, the primed phosphate-binding site, and the primed phosphorylation

**Figure 1:**
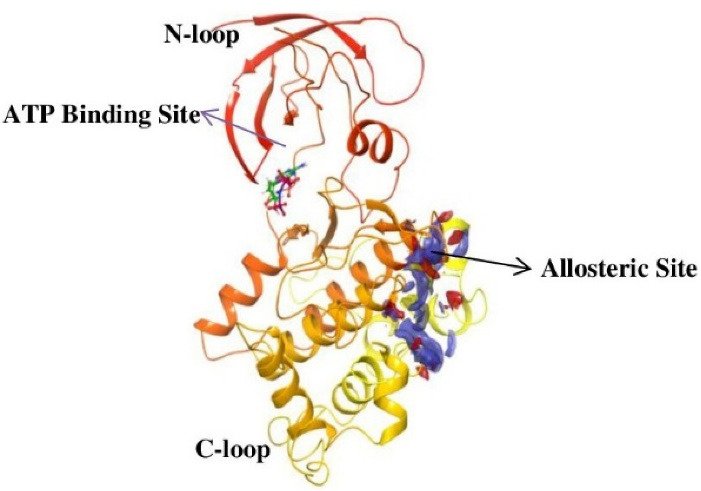
The cartoon representation of GSK-3β (PDB ID: 1PYX) showing the N-terminal and C-terminal region as well as the ATP binding site and possible allosteric drug binding site

 There are 12 exons in the human GSK-3β gene isoform. The human GSK-3β structure protein sequence is 433 amino acids in length and has a mass (Da) of 48 034, while the mouse GSK-3β Structure protein sequence is 420 amino acids long and has 46 710 mass (Da). The ATG start codon is found at exon-1, and the TAG stop codon is found in exon-12 in humans. The N-terminal domain, C-terminal domain, and kinase domain make up the structure of the GSK-3β protein. The kinase activity of GSK-3β has been inhibited by phosphorylation at SER9 in the N-terminal domain region. TYR216 phosphorylation in the activation site promotes GSK-3β phosphorylation of the substrate.^29^ GSK-3β is made up of two domains: an N-terminal β-strand domain contains residues from 25 to 138 with several antiparallel strands, and a C-terminal α-helical domain contains residues from 136 to 343. The ATP binding site shown in (Figure 1) is situated at the interface between the glycine-rich loop and the hinge domain. The activation loop contains amino acids from 200 to 226 as they are well-ordered and extend along the substrate-binding groove’s surface, whereas the C-terminal contains residues from 344 to 382 that are present outside the central kinase fold.^30^

 GSK-3 substrates with the prime phosphate at the site of n + 4 (n is the phosphorylation site) and has been phosphorylated more quickly than the substrate without the prime site. This means that in most cases, the prime site is four residues away (S/T-X-X-X-pS/pT). So, 5 and 3 amino acids priming at C-terminal has been explained.^31^ The residues at the N-terminal region of GSK-3β inhibit the enzyme from binding to the pseudosubstrate in the active site when it is phosphorylated at SER9. However, in both crystal (apo-GSK-3β crystal form A) (FRATtide & Sulphate ion crystal form FS) types of GSK-3β activation loop conformation is from 200 to 226 is very similar and includes the primed phosphate-binding pocket.^32^ Phosphorylation of the glycogen synthase by GSK-3β is unique and involved in previous phosphorylation by another protein kinase. E.g. phosphorylation by GSK-3 occurs once the SER656 of glycogen synthase has been phosphorylated by the casein kinase 2. GSK-3β then phosphorylates GS at SER652, SER648, SER644, and SER640 in a carboxy-terminal to the direction of the amino-terminal, with each subsequent phosphorylation dependent on the p + 4 position that has been being phosphorylated first.^33^ The periodicity of the phosphorylation sites (SER45, THR41, SER37, SER33, SER29) in the key Wnt target β-catenin suggests a related mechanism of sequentially primed phosphorylation is involved. GSK-3β mediated phosphorylation of THR231, a primed location that plays an important part in regulating the potential of tau to stabilize microtubules.^34^ GSK3β activity is inhibited by phosphorylation at SER9.^35^ The phosphorylation status of GSK3β influences its activity. GSK3β activity is increased when it is phosphorylated on TYR216 and inhibited when it is phosphorylated on SER9, and all of these activities are controlled by growth factor signalling.^36^ The simple triad of ARG96/ARG180/LYS205 is the phosphate-primed binding site of the substrates for GSK3β.

## Involvement of GSK-3 in AD

 The activity of GSK-3 is negatively regulated by the insulin signaling pathway, Wnt signaling pathway, and reelin signaling pathway. GSK-3 plays a pivotal role in the Hedgehog signaling cascade pathway. Insulin signaling activates glycogen synthase via dephosphorylation at a cluster of C-terminal amino acids such as SER641, SER645, SER649, and SER653, and phosphorylated by GSK-3α and GSK-3β.^37^

**Figure 2:**
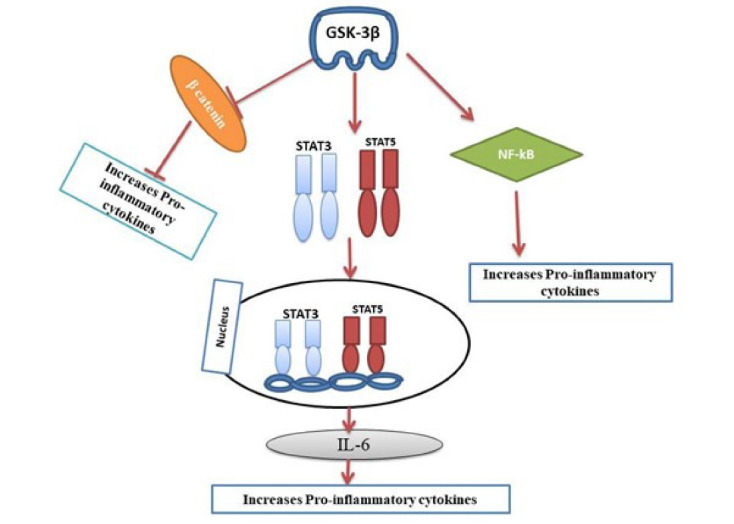
Up-regulation of Pro-inflammatory cytokines pathway in Alzheimer’s disease.GSK-3β down-regulates β-catenin and thereby inhibits the release of proinflammatory cytokines. GSK-3β also activates NF-kB signaling that results in increased production of proinflammatory cytokines

 GSK-3β has been recognized as a critical regulator of the inflammatory responses in immune cells, including microglia. Besides tau aggregation and Aβ, neuroinflammation is considered one of the major causes of AD.^38^ Aberrant GSK-3β can activate the pro-inflammatory state of microglia. The microglia are the immune cells of the brain, responsible for the production of pro-inflammatory cytokines resulting in neuroinflammation at the central nervous system (CNS).^39^ GSK-3 also induces neuroinflammation by activating toll-like receptors in monocytes and promoting glial fibrillary acid protein, a useful marker for astrogliosis.^40^ Thus, the inflammatory responses are overexpressed in AD.^41,42^ GSK-3β activation promotes the production of tumor necrosis factor-alpha, interleukin-6 (IL-6), IL-1 and also activates the transcription factors STAT3, STAT5, NF-κB signaling. The β-catenin is a direct target of GSK-3β phosphorylation and a transcriptional co-activator of the WNT signaling pathway^43^ (Figure 2). When β-catenin reaches the proteasomal degradation signaling or pathway then causes the decreased inhibition of especially NF-kβ signaling, so results in more production of NF-kβ inflammation responses. Whereas GSK-3β activates the transcription factors STAT3, and STAT5 and thereby activates IL-6 that leads to pro-inflammation. All these markers can increase the death of neurons and contribute to neurotoxicity.

**Figure 3:**
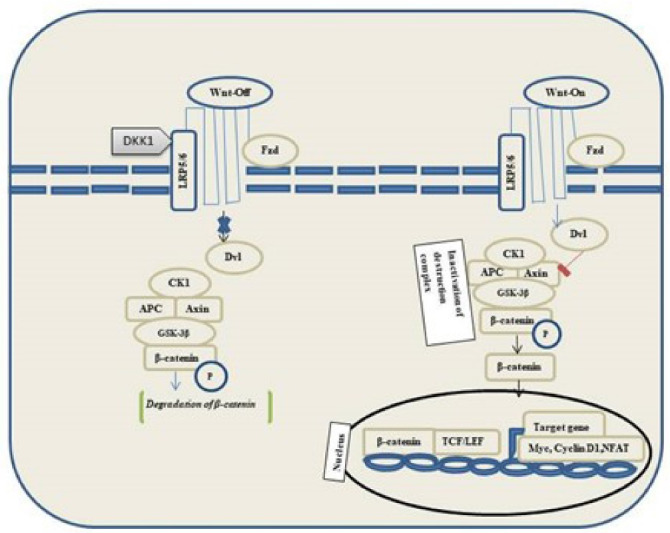
Overview of GSK-3β and β-catenin signaling pathways in the neural development of AD. Without Wnt, the level of β-catenin decreases, and it will not accumulate in the cytoplasm. Further the ubiquitination and the β-catenin send it to proteasomes for digestion. Wnt bind to FZ and LR65/6. Destruction complex function is disrupted, then β-catenin accumulates in the cytoplasm and then translocates into the nucleus. (Dvl, Dishevelled; DKK1, Dickkopf WNT signaling pathway inhibitor 1; LRP5/6, Low-density lipoprotein receptor-related protein; GSK-3β, Glycogen synthase kinase-3β; CK1, Casein kinase 1α; TCF/LEF, T cell factor/lymphoid enhancer-binding factor)

 Wnt binds to frizzled receptors of the family and low-density lipoprotein 5/6 in the canonical pathway. Destruction Complex functions are disrupted in the presence of Wnt. Phosphorylation of the destruction complex leads to the binding of Axin to the cytoplasmic tail region of lipoprotein 5/6. The components of destruction complex viz. Disheveled-Axin-DIX and PDZ domains inhibit the GSK-3 activity. Hence, the proteasomal destruction of β-catenin is inhibited, thereby leading to its accumulation in the cytoplasm followed by its translocation into the nucleus to function as a transcriptional co-activator of transcription factors to the lymphoid enhancement factor/ T-cell factor. In the absence of WNT, β-catenin does not accumulate in the cytoplasm because it is easily degraded by destruction complexes accompanied by ubiquitination, followed by proteasomal degradation of β-catenin (Figure 3).

 APP is further involved in the cleavage by the α-secretase complex consisting of ADAM-10 and 17. The α-secretase produces a peptide that is easily degraded and is non-amyloidogenic. APP is cleaved by the β-secretase (BACE-1) enzyme, which is amyloidogenic, and it is further cleaved by the γ-secretase complex. APP and presenilin-1 (PS1) are the catalytic components of γ-secretase, which tend to fibrilize, oligomerize, and gradually accumulate Aβ in AD’s brains. APP cleavage and PS1 activities are affected by increased GSK-3β activity resulting in increased amyloid-beta production and further deposited in AD brains. Activation of GSK-3β leads to neurodegeneration, specifically by encouraging the accumulation of Aβ. But PS1 is located in the endoplasmic reticulum, Golgi complex, and plasma membrane. PS1 is associated with beta-catenin/N-cadherin, which is involved in forming a trimeric synaptic complex of serine residues. Over-activation of GSK-3β can down-regulate the complex of PS1/beta- catenin/N-cadherin leading to neuronal loss and AD pathology.^44^

## Pockets of GSK-3β identified till today

 Using fpocket software, Palomo et al found seven pockets on the GSK-3β surface. They are 1. ATP binding site, 2. Substrate binding site, 3. Axin/FRAT tide binding site, whereas the 4^th^, 5^th^, and 7^th^ pockets are Allosteric sites^45,46^ and the 6^th^ pocket represents an orphan.^47^

 Pocket 1: This pocket was found in almost all crystal structures of GSK-3β and is well known as the ATP binding cavity that can be seen in many PDB structures (for example, 1Q3D co-crystallized with Staurosporine). Bertrand et al have postulated by docking studies that ATP competitive GSK-3β inhibitor Staurosporine (IC_50_: 15 nM) binds in the cleft formed between the N-terminal & C-terminal lobes of the enzyme this is popularly known as the hinge region. At the hinge region, hydrogen bone formation is observed with the N1 of staurosporine to the ASP133 carbonyl oxygen and O5 of staurosporine to the backbone nitrogen of VAL135. Staurosporine also makes a hydrophobic interaction with GSK-3β through the fuzed carbazole moiety forming major interactions with residues such as IIE62, GLY63, VAL70, GLY65, ALA83, TYR134, CYS 199, ASP133, ASN186, GLN185, LEU188 and ASP200.^48^

 Pocket 2: This pocket corresponds to the substrate-binding site, to validate this pocket Palomo et al used GSK-3β (PBD ID: 1H8F) co-crystallized with manzamine A. Manzamine A binds near the activation cavity to the enzyme formed by the residues such as ARG96, ARG180, and LYS205. To validate the substrate binding site Valle Palomo et al used GSK-3β (PBD I.D: 1Q4L) docked with 5-imino-1,2,4-thiadiazoles (ITDZs). PHE67, 89 to 95 loops are GSK-3β interacting sites with ITDZs.^49^

 Pocket 3: This pocket corresponds to the FRAT (frequently rearranged in T-cell lymphomas) tide binding site. It is present in the GSK-3β crystal structure (PDB ID: 1QMZ) with FRATtide. Whereas FRAT tide is a peptide, that corresponds to amino acids 188 to 226. For amino acid residues 198 to 222 of peptide (electron density is observed). Hydrogen bonds between residues TYR288 and GLU300 from GSK-3 and FRATtide the residues such as LEU212, IIE213, and LYS214.

 Pocket 4: This pocket corresponds to the allosteric binding site, to validate this pocket Chauhan et al used PDB ID: 4NU1, then the presence of glycerol with a hydrogen bond to ARG144 and some key interaction residues involved in binding they are GLU249, TYR140, ARG148, GLN185, TYR222, ARG220, TYR221, ARG144.^50^

 Pocket 5: This pocket corresponds to the allosteric site, whereas Bidon-Chanal et al, used GSK-3β (PDB ID: 1PYX) crystal structure for docking with palinurin. Then the binding site showed 2 differentiated cavities. Among them, cavity 1, the apolar cavity corresponds to the sesquiterpene moiety binding cavity containing amino acids such as VAL41,VAL40, PHE116, PHE115 and TYR117, 2^nd^ cavity where the tetronic ring binds to the polar residues such as TYR56, LYS86, SER118, and ASN129.^51^

 Pocket 6: This pocket corresponds to the orphan site, to validate this pocket Chauhan et al have used PDB ID: 1O9U. There are no reports of the heteroatoms and ligand binding to this pocket 6. The residues that line the cavity of the pocket 6 hinge region comprise GLU80, ARG113, ARG111, ASP133, VAL135, ASP190, and LYS197.

**Figure 4:**
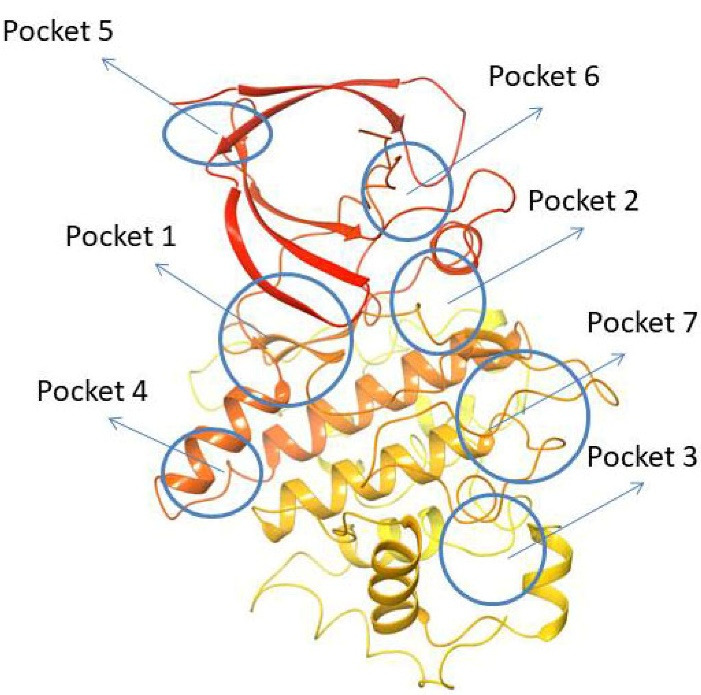
Cartoon representation of PDB I.D: 1PYX (GSK-3β protein) indicating all the possible drug-binding pockets

 Pocket 7: This pocket corresponds to the allosteric site, to validate this pocket Palomo et al used GSK-3β (PDB ID: 1PYX) docked with compound 1 (N′-Dodecanoyl-1-ethyl-4-hydroxy-2-oxo-1,2-dihydroquinoline-3-Carbohydrazide). Then ionic interaction between the hydroxyl group and ARG209 fixes compound 1 to the allosteric pocket. There is an interaction with SER236 and hydrophobic interactions found between the aliphatic chain of the compound with the hydrophobic site of the cavity THR330, ARG328 and PRO331.^52^ (Figure 4)

## Different classes of GSK-3β inhibitors

 Different classes of GSK-3 chemical inhibitors have been developed, such as competitive metal inhibitors, non-ATP competitive inhibitors, ATP-competitive inhibitors, peptide inhibition, allosteric inhibition and substrate competitive inhibitors.

###  Metal competitive GSK-3β inhibitors 

 The first inhibitor that inhibited GSK-3β specifically was lithium chloride (Table 1). GSK-3β was shown to have a decreased capacity to phosphorylate tau after being treated with lithium chloride via increased serum phosphorylation; lithium (Li^+^ ) serves as a GSK-3 competitive inhibitor in comparison to magnesium (Mg^2+^ ).^53^ Continuous lithium therapy improves cognition and memory levels in people with dementia; according to several clinical trials, lithium is a drug that has been used to cure bipolar disorder. But, in other studies, it has been included that patients treated with lithium, however, have found no such improvement in memory or reduction in tau phosphorylation in AD patients. Finally, in some aged population toxic side effects of lithium has been discovered. Beryllium (Be2 + ) is a most potent GSK-3β inhibitor than lithium chloride (Li^+^ ), competing for Mg^2+^ and ATP. Zinc (Zn2 + ) is a more potent GSK-3β inhibitor with an IC_50_: of ~15 μM, and it has also raised the cellular β-catenin levels.^54^ Zinc deficiency has been attributed to severe depression and cognitive impairment. In the SH-SY5Y neuroblastoma cell line, sodium tungstate (Na2WO4) has been shown to inhibit GSK-3β, resulting in the reduction of tau phosphorylation. Whereas Sodium tungstate has a low toxicity profile this is why a human clinical trial as an anti-obesity agent has been conducted.^55^

**Table 1:** Metal competitive inhibitors of GSK-3β in Alzheimer’s disease

**Drug**	**Structure**	**Class**	**Mode of action**	**References**
Lithium	Li^+^	Metal Competitive Inhibitor	Direct inhibition of GSK-3 by competition with Mg + and indirect inhibition through phosphorylation of SER9 with GSK-3β and phosphorylation of SER21 with GSK-3α	^56^

###  ATP-competitive inhibition 

 ATP competitive inhibitors interact with the amino acids, such as GLU97, LYS85 and ASP200, which are essential for ATP recognition. In addition, a hydrogen bond is formed at the hinge region with the backbone residues such as ASP133 and VAL135. Whereas most of the GSK-3β inhibitors are ATP competitive, their molecular weight is ( ≤ 600 g/mol), and ATP competitive inhibitors lack a high degree of kinase selectivity. 6-BIO, meridianins, amino pyrimidines, aryl indole maleimides, hymenialdisine, indirubins, pyrazines, phenyl methylene hydantoins, heteroaryl-pyrazole(3,4-b) pyridine, and paullones are examples of ATP-competitive inhibitors (Table 2). However, all these compounds have failed at the preclinical level because of their selectivity issue against the cyclin-dependent kinase-2 (CDK2) and a phylogenetically associated kinase that has a homology nearer to that ATP binding site of an enzyme. Compared to other CDK, GSK-3β has a greater ATP-binding site.^57^ Paullones is a highly effective and selective inhibitor of GSK-3α/β, and CDK5/p25 with IC_50_ values of 4 ± 80 nM and 20 ± 200 nM ranges, respectively. Paullones may thus be a promising new class of compounds for studying and treating neurodegenerative diseases.^58^

**Table 2:** ATP competitive inhibitors of GSK-3β in Alzheimer’s disease

**Drug**	**Structure**	**Mode of action**
6-BIO	**Figure:** 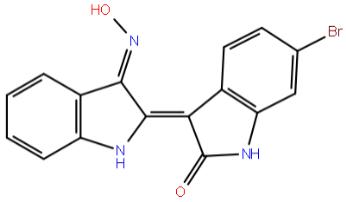	GSK-3β ATP competitive inhibition
Meridianins	**Figure:** 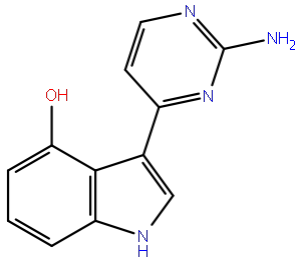	GSK-3β ATP competitive inhibition
Amino pyrimidines	**Figure:** 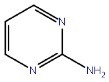	GSK-3β ATP competitive inhibition
Aryl indole maleimides	**Figure:** 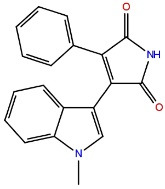	GSK-3β ATP competitive inhibition
Hymenialdisine	**Figure:** 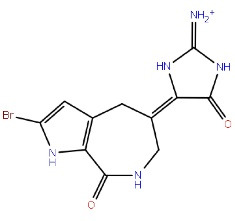	GSK-3β ATP competitive inhibition
Indirubins	**Figure:** 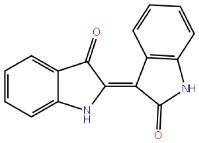	GSK-3β ATP competitive inhibition
Pyrazines	**Figure:** 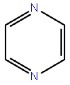	GSK-3β ATP competitive inhibition
Phenyl methylene hydantoins	**Figure:** 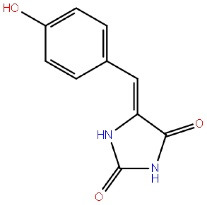	GSK-3β ATP competitive inhibition
Paullones	**Figure:** 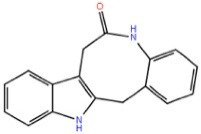	GSK-3β ATP competitive inhibition
Indirubin-3'-monoxime	**Figure:** 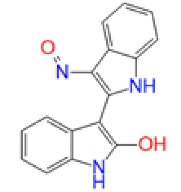	GSK-3 ATP-competitive inhibition
AR-A014418	**Figure:** 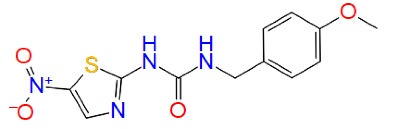	GSK-3 ATP-competitive inhibition
1-Azakenpaullone	**Figure:** 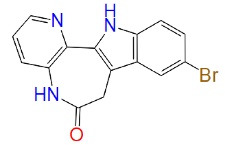	GSK-3β ATP competitive inhibition

###  Non-ATP competitive inhibition

 ATP competitive inhibition of GSK-3β has resulted in adverse effects, whereas non ATP competitive inhibitors don’t interact with the ATP intracellular concentration, so they have distinct pharmacological advantages. Thiadiazolidinediones (TDZD), benzothiazepinones, halomethylketones, and pyrazolotriazines are some examples of GSK-3β Non-ATP competitive inhibitors that have emerged in past years.^59^ The first irreversible inhibitor of GSK-3, halomethylketone, works by forming permanent sulfur–carbon bonds.^60^ Multiple biological functions are processed by pyrazolotriazines, including antiviral, antibacterial, and anti-inflammatory operations. An in-vitro model was used to assess tau hyperphosphorylation in cells. It can be considered new leads for more optimization in the area of AD pharmacotherapy.^61^

 Tideglusib (Table 3) is a GSK-3β non-ATP competitive inhibitor. It possesses anti-inflammatory and neuroprotective activities. It also reduces the phosphorylation of tau in human neuroblastoma cells and neural cell culture without causing any apoptosis. Tideglusib treatment improves cognitive deficits, phosphorylation of tau, amyloid accumulation, dystrophic neurites, neuronal loss, and astrocytic proliferation in transgenic mice dramatically.^62,63^

**Table 3:** Non–ATP competitive inhibitors of GSK-3β

**Drug**	**Structure**	**Mode of action**	**References**
NP-12 (Tideglusib/ NP031112)	**Figure:** 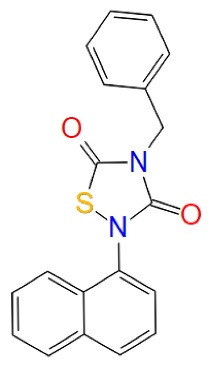	Non-ATP GSK-3β competitive inhibition	^64^
TDZD-8	**Figure:** 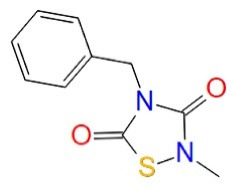	Non-ATP GSK-3β competitive inhibition	^65^

###  Allosteric inhibitors 

 Several medications with allosteric pathways in their targets have been accepted and widely distributed. Trametinib, a benzodiazepine used as a tranquilizer, and benzodiazepine Diazepam are some examples. However, there are no licensed drugs for AD-related targets yet, even though a large number of allosteric modulators have been identified. We further expect to enlighten knowledge in discovering the better advancement of science to find better AD treatments (Table 4). Palinurin (IC50 = 1.9 μM) binds to a particular allosteric cavity in the enzyme’s N-terminal domain, causing a conformational difference in the rich glycine loop that prevents ATP from reaching the active site and inhibiting GSK-3β action. Some of these cavities are used to represent potential allosteric sites, and it was also revealed a promising compound named VP0.7, a quinoline derivative with IC_50_ = 3.01 μM. Manzamine A was isolated from an Okinawan sponge of the *Haliclona* genus, which belongs to the Acanthostrongylophora family of marine sponges and Indo-Pacific sponges. Manzamine A (IC_50_ = 10.2 μM) was shown to be effective in reducing the phosphorylation of tau in a (human neuroblastoma) cell line following treatment, demonstrating its potential to penetrate cells and interfere with tau pathology in neurodegenerative disorders.^66^ Different -R groups, such as alkyl aryl, alkyl, and aryl, have been used to make benzothiazinones (BTOs). Compounds containing an ethyl or benzyl R group exhibited excellent action. These two chemicals, BTO-5s (IC_50_ = 10 μM) and BTO-5h (IC_50_ = 8 μM) inhibit GSK-3β in both allosteric and substrate-competitive ways. These GSK-3β allosteric inhibitors are still being studied, to optimize their drug-like properties and design new compounds based on their scaffolds. It’s worth noting that their paper is very useful in identifying the certain potential site of allosteric on the enzyme’s topography, which is one of the most critical current targets in the context of AD.^67^

**Table 4:** Allosteric inhibitors of GSK-3β in AD

**Drug**	**Structure**	**Class**	**Mode of action**	**References**
Palinurin	**Figure:** 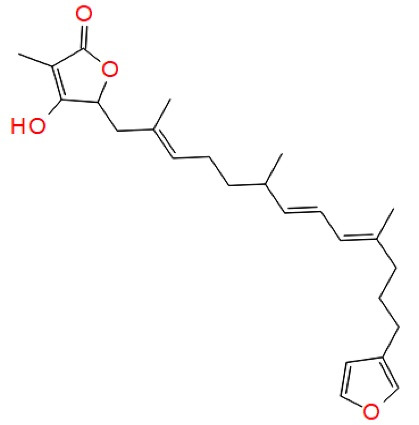	Furanosesquiterpenes	Allosteric GSK-3β inhibition	^51^
Manzamine A	**Figure:** 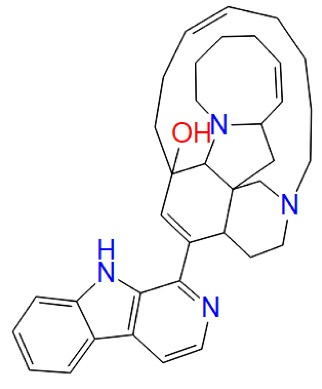	Manzamines	GSK-3 allosteric modulator	^68^
VP0.7	**Figure:** 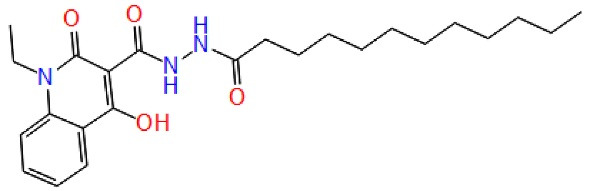	Quinoline derivative	Non-ATP GSK-3β competitive inhibition	^69^
Benzothiazinones (BTO-5h)	**Figure:** 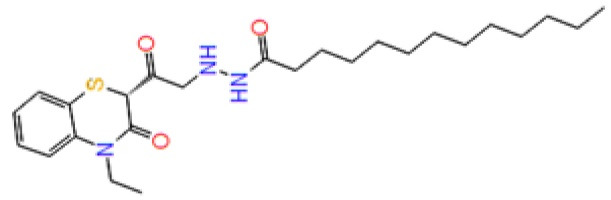	Benzothiazinones	GSK-3β allosteric inhibition	^21^

## Plant-based New therapeutics and Combinations of known drugs with GSK-3β inhibitors

 Ginger, the rhizome of the plant *Zingiber officinale,* is especially used by the Asian and African populations. Ginger is also used in traditional medicines. Whereas 6-gingerol, the phenolic compound, which is found in the rhizomes of ginger possesses anti-tumor activity, anti-inflammatory, antioxidant properties, and neuroprotective activity against the cell apoptosis induced by amyloid-beta through its anti-oxidative role. According to the rodent study by Zeng et al, an extract of ginger root may reverse behavioral disorders and avoid AD-like symptoms. Whereas 6-gingerol ( ≥ 98% purity) was obtained from the industry (Shanghai Fu life). It was found to substantially inhibit the expression of phosphorylated GSK-3β at SER9 phosphorylated Akt at SER473, which is caused by Aβ1-42. Then 6-gingerol may enhance the neuroprotective activity through AKT/GSK-3β signaling pathway. So, it might be used for the treatment of AD.^70^

 Morin is a flavonoid found in Osage orange (*Maclura pomifera*), guava, onion, and apple. It was first isolated from mulberry figs and old fustic heartwood. It inhibits the development of Aβ-fibrils, and it also possesses antioxidant, anticancer, anti-inflammatory, cytoprotective, and anti-amyloidogenic agents. Both *in-vivo and in-vitro* studies have shown that morin inhibits GSK-3β induced tau phosphorylation. The morin which was procured from Sigma Chemical was used for intraperitoneal administration to 3xTg–AD mice resulting in a reduction of hyperphosphorylation of tau.^71^ According to this article, the author concluded that morin prevents amyloid beta-induced hyperphosphorylated tau in human neuroblastoma cells. However, molecular processes and cognitive deficiencies in AD animal models will need further clinical research.^72^

 Berberine is an isoquinoline alkaloid derived from the *Rhizoma coptidis*. It is present in the roots, bark, and stems. Berberine possesses anti-inflammatory, cardioprotective, neuroprotective, anti-tumor, anti-obesity, and antimalarial properties. Because of inhibition of Akt/GSK3/apoptotic/ERK1/2 survival signaling pathway and Caspase-3 activity and JNK have been linked to berberine-mediated neuroprotection effect after ischemia. Berberine also reduces oxidative stress and inflammation in AD patients’ brains. The Berberine was procured from (St. Louis, MO, USA) Sigma-Aldrich.^73^

 Furthermore, research is required to determine the function of berberine in reducing all the risk factors and pathologies associated with AD. Berberine might be useful in reducing AD by lowering the pathogenesis of intracellular neurofibrillary tangles and amyloid plaques extracellularly. Long-term treatment with berberine reduced hyperphosphorylation of tau via the AKT/GSK-3β pathway, and berberine improved cognitive dysfunction in 3xTg-AD mice.^74^

 Arctigenin is a phenylpropanoid dibenzyl butyrolactone lignin. Arctigenin possesses anti-inflammatory, antioxidant, antibacterial, anti-tumor, anti-influenza, and hypoglycaemic effects. Arctigenin inhibits hyperphosphorylation of tau in the hippocampus and effectively protects against memory and learning deficits. Arctigenin with (purity ≥ 98%) was prepared by their laboratory, has elevated the GSK-3β (SER9) phosphorylation, phosphorylation of PI3Kp85, and phosphorylation level of Akt/Ser and suppresses the PI3K/Akt activation level. Qi et al have shown that arctigenin protects against ICV A1–42-induced memory and also learning deficits by lowering hyperphosphorylation of tau in the GSK-3β/PI3K/Akt dependent signaling pathway. Some findings indicate that arctigenin inhibits Aβ-induced hyperphosphorylation of tau in the PI3K/Akt/GSK-3β signaling. As a potential mechanism, then the PI3K/GSK-3β/Akt dependent pathways could be inhibited. Based on all these findings, arctigenin possesses anti-tau hyperphosphorylation activity, and this Arctigenin could be a target for AD treatment (Table 5).^75^

**Table 5:** Plant-based inhibitors of GSK-3β

**Drug**	**Structure**	**Reference**
Morin	**Figure:** 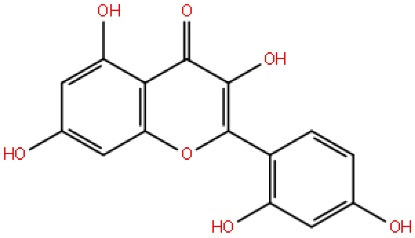	^71^
Berberine	**Figure:** 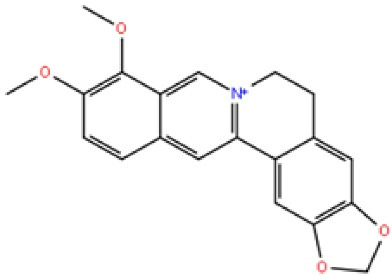	^74^
Arctigenin	**Figure:** 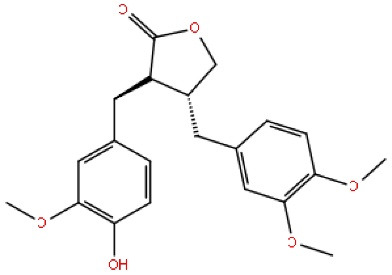	^75^

 Salvianolic acid B is the most common traditional Chinese herb. It is used to treat heart disease, stroke, cerebrovascular disorder, renal damage, AD, and Parkinson’s disease. Salvianolic acid B has antioxidant and anti-free radical properties, and it can be used to treat neurodegenerative diseases.^76^ In human neuroblastoma, SH-SY5Y cell line Aβ-induced neurotoxicity is minimized by the *Salvia miltiorrhiza* (Danshen). It inhibits the tau-related kinase GSK-3β activity directly, which reduces the hyperphosphorylation of tau and possesses a better chance of treating AD.^77^

## Preclinical & clinical studies of GSK-3β inhibitors

 In the last several years, a significant effect has been made by synthesizing a large number of selective and potent inhibitors of GSK-3, among them, some have shown well in vivo efficacy in various AD animal models.

 Tideglusib (Table 6) is a GSK-3β non-ATP competitive inhibitor (NP031112 or NP12). It was developed for the treatment of AD. In vivo studies that tideglusib reduced amyloid-beta, tau phosphorylation, gliosis, and neuronal loss also reverse a spatial memory deficit in transgenic mice. Patients who took the drug tideglusib showed a slower rate of brain atrophy development. But U.S. FDA discontinued tideglusib from clinics for the treatment of AD.^78,79^

**Table 6:** Preclinical studies involving GSK-3β inhibitors

**Compounds**	**Species**	**Model**	**Effects**	**References**
Lithium	Rats	Administered amyloid-beta into the intra-hippocampal region	Interruption of spatial memory problems	^80^
Indirubin-3-monoxime	Mice	Double transgenic mice App23/ps45	Both Spatial learning and memory deficits have been improved	^82^
AR-A014418	Mice	Double transgenic mice App23/ps45	Improvement of both spatial learning and memory deficits	^83^
AZD 2858	Rodent	Transgenic model overexpressing GSK3	Inhibition of hyperphosphorylation of tau in rodent brain hippocampus and gliosis (neurodegeneration marker)	^84^
AZD1080	Sprague–Dawley rats	Normal Rodent brain following in vivo administration of the acute dose of AZD1080 (1, 3, or 10 mol/kg) p.o.	Inhibition of tau phosphorylation and reduction of the phosphorylated to total Glycogen Synthase ratio	^85^
NP-12	Mice	Human mutant APP and tau were co-expressed in aged double transgenic mice.	Improvement of both Spatial learning and memory deficits	^86^

 AZD 1080 was developed by AstraZeneca for AD treatment. AZD 1080 is a GSK-3β ATP competitive inhibitor. AstraZeneca 2008 discontinued the development of AZD 1080 in phase 1 trial in the United Kingdom, because of disarrangement of the cytoskeleton, neurofibrillary tangles formation, and leads to the death of neurons.^81^

## Discussion

 GSK-3β plays a part in the crucial role of memory, neuronal fate determination, and behavior. GSK-3β has emerged as one of the potential drug targets for an array of diseases ranging from cancer to diabetes, polycystic kidney disease, and AD. GSK-3β has also been implicated in AD, whereas the increased activity of GSK-3β that leads to hyperphosphorylation of tau protein which can promote neurogenesis, impaired synaptic plasticity apoptosis, and hyperexcitability in the hippocampus. The allosteric process is commonly favored by other conventional processes because it has a reduced chance of causing side effects. Because of the allosteric mechanism, the ligand will have to contend with the endogenous substrate on the active site.

 Allosteric is a biologically important feature of many enzymes. A compound binding at an allosteric site may affect the conformational transition. Owing to their interaction in particular and special binding sites, allosteric ligands interact with substrates in a more selective way, which may be beneficial in resolving drug-induced resistance. Furthermore, when treating chronic or long-term disorders like AD, high selectivity and mild regulation of certain targets lead to better outcomes.

## Conclusion

 AstraZeneca described ATP-competitive GSK3β inhibitors such AZD1080 as orally active & brain permeable GSK-3 inhibitors that inhibited human GSK-3β in the nanomolar range, however owing to nephrotoxicity in phase 1 clinical study targeting AD, further development of this inhibitor was terminated. Similarly, ATP-competitive GSK-3β inhibitors are less selective and have shown more side effects in clinical studies. Allosteric modulators of the kinase proteins are likely to be more selective, but there is no information related to *in vivo *studies based on Allosteric GSK-3β inhibitors.

 The selectivity, potency, and safety of the GSK-3β inhibitors are vital for consideration as a potential therapeutic alternative. Several research works have been carried out in the search for novel alternatives. Allosteric GSK-3β inhibitors are being pursued as more selective and safer drugs as alternative inhibitors. Allosteric inhibitors have a binding site that is different from that of the ATP pocket. GSK-3β Allosteric inhibitors have more advantages than other GSK-3β inhibitors and could open new avenues.

## Acknowledgments

 These authors acknowledged the infrastructural support from the “Department of Pharmacology”, Manipal College of Pharmaceutical Sciences, ‘Manipal Academy of Higher Education (MAHE), Manipal, Pincode: 576104, India. The authors would like to thank “Dr. TMA Pai” for the scholarship.

## Competing Interests

 The authors declare that they were no conflict of interest.

## Ethical Approval

 Not applicable.
